# Gastroenteritis Related Seizure with or without Fever: Comparison Clinical Features and Serum Sodium Level

**Published:** 2019

**Authors:** Farhad HEYDARIAN, Elham BAKHTIARI, Shima BADZAEE, Mohammad HEIDARIAN

**Affiliations:** 1 Department of Pediatrics, Faculty of Medicine, Mashhad University of Medical Sciences, Mashhad, Iran; 2 Research Center for Patient Safety, Mashhad University of Medical Sciences, Mashhad, Iran; 3 Ghaem Hospital, Mashhad University of Medical Sciences, Mashhad, Iran; 4 Biological Sciences Student, California State University, East Bay, Hayward, California, USA

**Keywords:** Fever, Gastroenteritis, Child, Sodium, Seizure

## Abstract

**Objective:**

This study investigated the clinical characteristics and serum sodium level in children with gastroenteritis related seizure with or without fever.

**Materials & Methods:**

This clinical study was performed in Ghaem Hospital, Mashhad University of Medical Sciences, Mashhad, Iran from 2007 to 2014. Overall, 165 patients aged 6-60 months with gastroenteritis related seizure were studied. Demographic, seizure and gastroenteritis characteristics and laboratory findings were recorded.

**Results:**

Among the 165 children 47.3% were female. Vomiting was 2.7±2.6 and 3.9±1.9 times in febrile and afebrile group. Duration of diarrhea was 1.8±1.8 days and 2.1±1.3 days in febrile and afebrile groups (p=0.014). 36% in febrile group and 6.4% in afebrile group experienced seizure within the first 24 h of gastroenteritis (*P*<0.001). Seizure in 99.1% in febrile and 93.6% in afebrile group was generalized (*P*>0.05). Seizure was more than 5 min in 51.4% in febrile and 57.4% in afebrile groups (*P*>0.05). Drowsiness after seizure was seen in 72.9% and 60% in febrile and afebrile group respectively (*P*>0.05). The serum level of sodium was 137.6±3.98 mEq/L and 138.5±3.78 mEq/L in febrile and afebrile groups (*P*>0.05). 26.3% in febrile group and 8.5% in afebrile group had hyponatremia (*P*=0.012). There was no difference in seizure duration between hyponatremic patients and others (*P*>0.05).

**Conclusion:**

Type, duration of seizure and drowsiness after seizure had not any difference in febrile and afebrile cases. Vomiting and duration of diarrhea before admission was lower in febrile group. Seizure within the first 24 h of gastroenteritis was higher in febrile group. Mild hyponatremia in febrile group was higher than afebrile group. No difference in duration of seizure was detected between hyponatremic patients and others.

## Introduction

Gastroenteritis (GE) is known to be capable of inducing benign afebrile seizure in infants and young children (1). The benign convulsion associated with mild gastroenteritis (CwG) for the first time (2). Occurrence of convulsion in the setting of gastroenteritis has long been documented as a complication of bacterial infections including shigellosis and Campylobacter (3-5). Recently, gastroenteritis related seizure have been reported, frequently (6-8). Regarding the difference between febrile and afebrile patients, afebrile seizures were included in many reports, while febrile seizures during gastroenteritis were considered in few studies (6, 7, 9, 10). 

this study was conducted, to evaluate the clinical features and serum sodium level of children with gastroenteritis related seizure with or without fever.

## Materials & Methods

This clinical study was performed in Ghaem Hospital, Mashhad University of Medical Sciences, Mashhad, Iran from 2007 to 2014. 

The study participants included 165 admitted children, aged between 6-60 months with gastroenteritis related seizure. Sample size study populations of 165 patients were considered appropriate to achieve a reasonable statistical analysis. The diagnosis of gastroenteritis was documented based on the clinical examination and symptoms.

Patients with severe dehydration, hypernatremia (serum sodium level >150 mEq/L), hyponatremia (serum sodium level<130 mEq/L), acute or chronic renal disease, meningitis, hypocalcemia, hypoglycemia, febrile convulsion or epilepsy history were excluded. 

This study was approved by the Ethics Committee of Mashhad University Medical Sciences. 

**Sample size**

**Analysis**

Statistical analysis was performed using SPSS windows program Ver. 16 (Chicago, IL, USA). All experimental values are presented as Means ± standard deviation (SD). The comparison between groups was done by t-test or nonparametric equivalent. *P*-values less than 0.05 was considered statistically significant.

## Results

***Demographic***

Among the 165 included children, 118 patients had fever and 47 patients had afebrile seizure. In febrile group, 47 cases (39.8%) were female and 71 cases (60.2%) were male with the average age of 23.2±15.58 months. In afebrile group, 31 cases (66%) were female and 16 cases (34%) were male with the average age of 29±13.91 months. The difference between groups in sex and age was significant (*P*=0.002 and *P*=0.01, respectively). The average weight in febrile and afebrile groups was 11.3±3.4 kg and 12±2.84 kg, respectively (*P*>0.05). One patient in febrile group (0.8%) and one patient in afebrile group (2.1%) had a family history of epilepsy, as well as 8 patients (6.8%) in febrile group and 4 patients (8.5%) in afebrile group, had a history of febrile seizure in their families (*P*>0.05). 

***Seizure characteristics***

Comparison of seizure characteristics showed that 40 patients (36%) in febrile group and 3 patients (6.4%) in afebrile group experienced seizure within the first 24 h of GE (*P*<0.001). Generalized seizure was detected in 110 cases (99.1%) in febrile group and 44 cases (93.6%) in afebrile group (*P*>0.05). Overall, 92 patients (82.9%) in febrile group and 35 patients (74.5%) in afebrile group experienced only one episode of seizure (*P*>0.05). Duration of the seizure attack was more than 5 min in 57 patients (51.4%) in febrile group and 27 (57.4%) patients in afebrile group (*P*>0.05). Drowsiness after seizure was observed in 86 patients (72.9%) in febrile group and 27 patients (60%) in afebrile group (*P*>0.05) (Table 1). 

***Gastroenteritis characteristics***

Comparison of GE characteristics showed that the frequency of vomiting before hospitalization in febrile and afebrile patients was 2.7±2.6 and 3.9±1.9 times respectively (*P*=0.001). The frequency of diarrhea before hospitalization was 5.5±3.89 times in febrile group versus 5.5±2.86 times in afebrile group (*P*>0.05). The duration of diarrhea before hospitalization was 1.8±1.8 days in febrile group and 2.1±1.3 days in afebrile group (*P*=0.014). The duration of diarrhea within hospitalization in febrile and afebrile groups was 2.1±1.4 days and 2.3±1.4 days, respectively (*P*>0.05). The duration of hospitalization in febrile and afebrile groups was 4.1±1.9 days and 4.1±1.6 days respectively (*P*>0.05). 

**Laboratory tests**

The mean of serum level of sodium was 137.6±3.9 mEq/L in febrile group and 138.5±3.78 mEq/L in afebrile group (*P*>0.05). Thirty one patients (26.3%) in febrile group and 4 patients (8.5%) in afebrile group had hyponatremia at admission (the sodium level less than 135 mEq/L) (*P*=0.012). 

There was not any significant correlation between serum level of sodium and gastroenteritis characteristics including number of vomiting, frequency of diarrhea and duration of diarrhea before hospitalization (*P*>0.05). 

Moreover, in 57.1% of hyponatremic patients and 52% of non hyponatremic patients, the duration of seizure was more than 5 min (*P*>0.05). The duration of seizure in 17 hyponatremic patients in febrile group and 3 hyponatremic patients in afebrile group was more than 5 min (*P*>0.05).

## Discussion

Overall, 165 patients with gastroenteritis related seizure were studied evaluating the clinical features and serum level of sodium in febrile and afebrile cases. The afebrile patients had more vomiting episodes before hospitalization as well as more duration of diarrhea from onset of gastroenteritis until hospitalization compared with febrile group. However, seizure within the first 24 h of gastroenteritis was higher in febrile cases. In a study on children with febrile and afebrile seizure associated with rotavirus gastroenteritis, the duration of gastrointestinal symptoms before the onset of seizure was significantly shorter in febrile group compared with afebrile group (1). A study on epidemiologic and clinical characteristics of 24 patients with CwG, the female/male was 2.8 (11). Our study showed that in afebrile group female/male was 2 that is compatible with Dura-Trave report (11). 

**Table 1:** Seizure characteristics of 165 patients with febrile and afebrile seizures associated with gastroenteritis

	**Febrile group (%)**	**Afebrile group (%)**	***P *** **value**
**Seizure type**			
**generalized** **focal nest**	110(99.1) 1(0.9)	44(93.6) 2(4.3)	0.11
**Frequency of seizure episodes before referral to hospital**			
**1 time** **2 times** **3 times** **More than 3 times**	92(82.9) 14(12.6) 3(2.7) 2(1.8)	35(74.5) 7 (14.9) 3(6.4) 2(4.2)	0.19
**Duration of the seizure attack (min)**			
**Less than 5** **More than 5**	54 (48.6) 57(51.4)	20(42.6) 27(57.4)	0.48
**Seizure within the first 24 h of GE**	40(36)	3(6.4)	<0.001*
**Dizziness after seizure**	86(72.9)	27(60)	0.11

* Significant difference in statistical analysis

In a study on 59 febrile and afebrile children with gastroenteritis related seizure, there was no significant difference between groups in the age, family history of FS, clinical symptoms and frequency of convulsion (12). The results of present study are consistent with another study in family history, clinical symptoms, and frequency of convulsion but not in age. The age of afebrile patients was greater than febrile patients. 

There was a positive family history of epilepsy and FS in 6% and 7% of Afebrile FS children respectively (10). In present study, there was a familial history of epilepsy and FS in 0.8% and 6.8% of afebrile patients. The duration of seizures as 5 min or more was longer in FS group in comparison with AFS group (34% versus 11%) while in our study was 51% versus 57% (12). The difference may be due to that we included the seizures which occurred before hospitalization. 

Overall, 86% of patients had tonic-clinic generalized seizures and 70% had FS (13). Fever had not any significant effect on duration and characteristics of seizure. (13). In this study, there was no significant difference in type and duration of seizures between febrile and afebrile group. According to present study, there was not any significant difference between serum sodium levels in two groups, but relative hyponatremia was significantly more in febrile group. It may be due to excess of Anti-diuretic hormone  (ADH) secretion. 

In the present study, there was not any significant difference in seizure duration between hyponatremic patients and others. Relative hyponatremia had not any role in duration of seizure in our cases. Heydarian reported that there was no relationship between serum sodium changes and seizure occurrence in children with hypernatremia (14). However, hyponatremia affected the seizure characteristics, hyponatremic children experienced longer duration of seizure compared with normal subjects (13).

**In conclusion, **most clinical features including type, duration of seizure and drowsiness after seizure had not any significant difference in febrile and afebrile gastroenteritis related seizure. However, frequency of vomiting and duration of diarrhea before admission was significantly lower in febrile group. Seizure within the first 24 of gastroenteritis was significantly higher in febrile group. Although there was not any significant difference between serum levels of sodium between two groups, relative hyponatremia occurs more in febrile cases. 
